# Phenylephrine increases cardiac output by raising cardiac preload in patients with anesthesia induced hypotension

**DOI:** 10.1007/s10877-018-0126-3

**Published:** 2018-03-22

**Authors:** A. F. Kalmar, S. Allaert, P. Pletinckx, J.-W. Maes, J. Heerman, J. J. Vos, M. M. R. F. Struys, T. W. L. Scheeren

**Affiliations:** 1 Department of Anesthesia and Critical Care Medicine, Maria Middelares Hospital, Buitenring Sint-Denijs 30, 9000 Ghent, Belgium; 2 Department of Surgery, Maria Middelares Hospital, Ghent, Belgium; 3 Department of Anesthesiology, University of Groningen, University Medical Center Groningen, Groningen, The Netherlands; 4 Department of Anesthesia, Ghent University, Ghent, Belgium

**Keywords:** Hemodynamic monitoring, Fluid responsiveness, Phenylephrine, Cardiac output, Pulse pressure variation

## Abstract

Induction of general anesthesia frequently induces arterial hypotension, which is often treated with a vasopressor, such as phenylephrine. As a pure α-agonist, phenylephrine is conventionally considered to solely induce arterial vasoconstriction and thus increase cardiac afterload but not cardiac preload. In specific circumstances, however, phenylephrine may also contribute to an increase in venous return and thus cardiac output (CO). The aim of this study is to describe the initial time course of the effects of phenylephrine on various hemodynamic variables and to evaluate the ability of advanced hemodynamic monitoring to quantify these changes through different hemodynamic variables. In 24 patients, after induction of anesthesia, during the period before surgical stimulus, phenylephrine 2 µg kg^−1^ was administered when the MAP dropped below 80% of the awake state baseline value for > 3 min. The mean arterial blood pressure (MAP), heart rate (HR), end-tidal CO_2_ (EtCO_2_), central venous pressure (CVP), stroke volume (SV), CO, pulse pressure variation (PPV), stroke volume variation (SVV) and systemic vascular resistance (SVR) were recorded continuously. The values at the moment before administration of phenylephrine and 5(T_5_) and 10(T_10_) min thereafter were compared. After phenylephrine, the mean(SD) MAP, SV, CO, CVP and EtCO_2_ increased by 34(13) mmHg, 11(9) mL, 1.02(0.74) L min^−1^, 3(2.6) mmHg and 4.0(1.6) mmHg at T_5_ respectively, while both dynamic preload variables decreased: PPV dropped from 20% at baseline to 9% at T_5_ and to 13% at T_10_ and SVV from 19 to 11 and 14%, respectively. Initially, the increase in MAP was perfectly aligned with the increase in SVR, until 150 s after the initial increase in MAP, when both curves started to dissociate. The dissociation of the evolution of MAP and SVR, together with the changes in PPV, CVP, EtCO_2_ and CO indicate that in patients with anesthesia-induced hypotension, phenylephrine increases the CO by virtue of an increase in cardiac preload.

## Introduction

The ultimate goal of hemodynamic management is to maintain adequate tissue oxygen delivery to the different end-organs [1]. Surgical patients often suffer relative hypovolemia owing to a combination of epidural analgesia, general anesthesia and patient positioning. The principal aim of goal-directed fluid therapy is to optimize the position of the heart on the Frank–Starling curve by increasing cardiac preload. This is conventionally pursued by administration of fluids to increase total blood volume and can improve patient outcome by reducing postoperative complications and length of hospital stay [2].

Phenylephrine is a direct α-adrenergic receptor agonist, predominantly α_1_, increasing the systemic vascular resistance (SVR) and arterial pressure [3]. While venous α_1_ receptor activity is acknowledged scientifically, in most clinical conditions, phenylephrine is considered to increase cardiac afterload but not cardiac preload. In patients with life threatening septic shock, norepinephrine has been shown to increase venous return in case of preload dependence [4, 5]. Nevertheless, while a beneficial effect of phenylephrine on the blood pressure is obviously well known, phenylephrine is conventionally postulated to have no effects on CO, but owing to an increase in afterload, would in most cases even decrease CO. In contrast, however, while an increase in left ventricular afterload may decrease stroke volume (SV) and thus cardiac output (CO) [6], the α_1_-adrenergic receptor stimulation—either by phenylephrine or norepinephrine—also decreases venous capacitance, which could in turn increase cardiac preload and SV [3]. It has been shown in pigs that the impact of phenylephrine on the CO is related to preload dependency [7]. When the heart is preload independent, phenylephrine induces on average a decrease in CO, whereas when the heart is preload dependent, it induces on average an increase in CO [6]. We hypothesize that in preload-dependent patients due to pronounced relative hypovolemia induced by combined epidural and general anesthesia, in leg-down position, phenylephrine may increase cardiac preload by virtue of centralisation of blood volume.

The aim of this study was to differentiate the chronicity of the changes in cardiac preload and CO after a single administration of phenylephrine and to assess the ability of advanced hemodynamic monitoring to quantify these changes through different hemodynamic variables.

## Methods

**Fig. 1:**
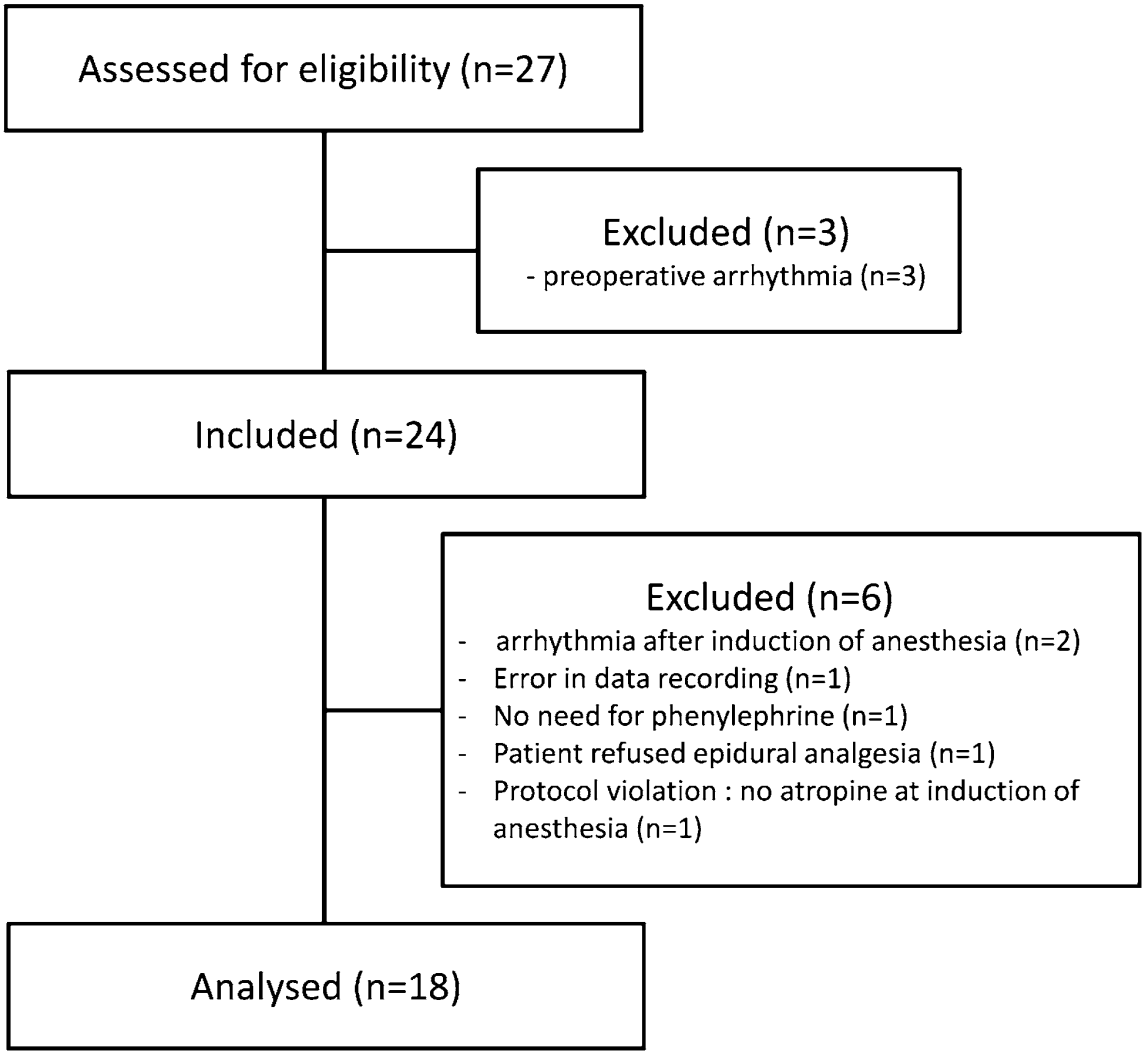
Flow chart of the patients’ inclusion and analysis

This prospective interventional study was approved by the institutional review board and was registered at clinicaltrials.gov (NCT:02739399; PI Dr. A Kalmar; April 15, 2016). The manuscript adheres to the applicable STROBE guidelines. After written informed consent was obtained, a total of 24 adult patients, scheduled for elective laparoscopic sigmoidectomy were included (Fig. 1). Patients with cardiac arrhythmia or a contraindication for atropine or phenylephrine administration were excluded.

### Study protocol

No premedication was administered. Upon arrival in the operating theatre, a peripheral intravenous line was inserted and an epidural catheter was placed. After adequate pre-oxygenation, induction and maintenance of anesthesia was pursued by target-controlled total i.v. anesthesia with propofol and remifentanil. At the start of induction of anesthesia, intravenous methylatropine 0.5 mg and epidural levobupivacaine 50 mg were given. After the administration of *cis*-atracurium and endotracheal intubation, the patients’ lungs were mechanically ventilated in the volume control mode (tidal volume: 8 mL kg^−1^) with an O_2_/air mixture (F_i_O_2_ 0.6) and a PEEP of 4 cm H_2_O. During the study period, the ventilatory settings were unchanged. A radial artery was cannulated using a 20 G catheter and connected with a disposable ProAQT transducer from the Pulsioflex monitor (Maquet, Rastatt, Germany)—all measurements were conducted with the same Pulsioflex monitor and automatically calibrated. This minimal invasive device enables calculation of the mean arterial blood pressure (MAP), heart rate (HR), SV and CO using pulse contour analysis of the arterial pressure curve. In addition, it calculates pulse pressure variation (PPV) and stroke volume variation (SVV) as measures of cardiac preload dependency as well as SVR as one of the determinants of left ventricular afterload. Next, a central venous catheter was placed for continuous recording of the central venous pressure (CVP) and the patient was positioned in preparation for surgery. All pressure transducers were located at the level of the right atrium before initiation of the study period. During the subsequent period prior to surgical stimulus, when MAP dropped below 80% of the awake state baseline value for > 3 min, a bolus of phenylephrine 2 µg kg^−1^ was administered. From 3 min before until 13 min after phenylephrine administration, patient positioning was left unaltered, and no other medication or fluid was administered.

### Data registration and analysis

All anesthetic data were collected on the anesthesia monitor (Philips MP70; Philips, Eindhoven, The Netherlands) and recorded at 0.2 Hz for subsequent offline analysis. The electronic data were imported into Microsoft Excel 2010® (Microsoft, Redmond, USA) for analysis. In addition to the recorded variables, an analogue of the mean systemic filling pressure (Pmsa) was calculated using the formula Pmsa = a × CVP + b × MAP + c × CO, in which a = 0.96, b = 0.04 and c is calculated according to anthropometric data [8]. Subsequently, resistance to venous return (RVR) was calculated as RVR = (Pmsa − CVP). CO^−1^ and pressure for venous return (Pvr; as a measure of venous return) was calculated as Pvr = Pmsa − CVP.

The evolution of the absolute values and of the changes relative to baseline (T_−1_) was analyzed from 1 min before induction (T_−1_) of anesthesia until the relative steady state was achieved for all study variables after 10 min (T_10_). All measurements for the study were performed before surgery commenced.

### Statistical analysis

Assuming a normal distribution of the CO data, we considered a mean increase in CO of 15% to be clinically relevant (estimated SD of 0.7 L min^−1^, based on pilot data). To detect this difference with an α-error of 0.05 and a power of 0.95, a total of 17 patients is needed [9]. A supplemental 40% of patients were included to anticipate exclusions, making a total of 24 patients.

Normality and homoscedasticity were tested with the Kolmogorov–Smirnov test and modified Levine test, respectively. Continuous data are expressed as mean(SD). For statistical analysis and visualization, the individual patient measurements were synchronized at the moment (T_0_) of 10 mmHg increase in MAP after phenylephrine administration.

**Fig. 2:**
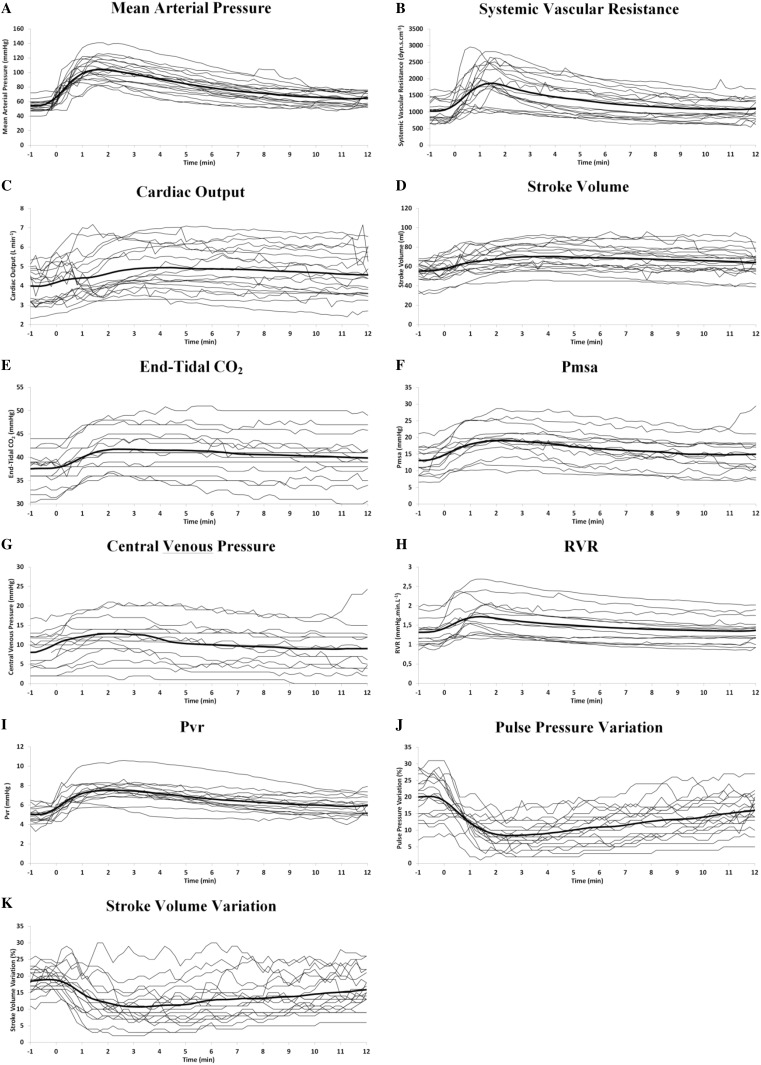
The evolution of individual patient variables. The evolution in individual patients (thin lines) and average (thick line) values of the main preload-dependent variables over the period from 1 min before till 12 min after the increase in initial blood pressure following the administration of phenylephrine. All measurements are synchronized at the moment (T_0_) of 10 mmHg increase in MAP following phenylephrine administration

**Fig. 3:**
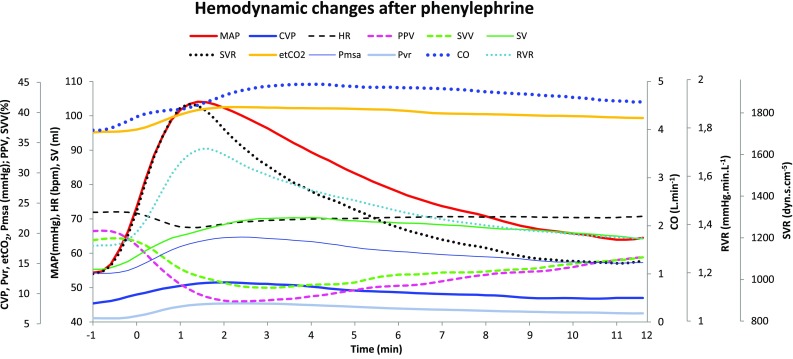
The course of the hemodynamic variables after administration of phenylephrine. The MAP, CVP, HR, PPV, SVV, SV, SVR, end-tidal CO_2_-concentration (EtCO_2_), mean systemic filling pressure (Pmsa), CO and resistance to vascular return (RVR) are shown. The graphs are the averages of the individual patient measurements, synchronized at the moment (T_0_) of 10 mmHg increase in MAP after phenylephrine administration

For visual assessment of systematic changes of the main variables, the evolution of the individual patient values, as well as the evolution of the mean value were depicted in Fig. 2. For comprehensive visualization of the chronicity and interaction of the different variables, the average values of all the studied variables were shown in Fig. 3.

The absolute values of the analysed variables were determined at 1 min before the increase in MAP (T_−1_), and 5(T_5_) and 10(T_10_) min afterwards. Results were subject to the general linear model repeated measures ANOVA with Bonferroni adjustment. All statistics were performed using S-PLUS 8.0 (TIBCO Software Inc., Palo Alto, CA, USA) and SPSS 23.0 (SPSS Inc., Chicago, IL, USA). Significance was set at P < 0.05.

## Results

Six patients were excluded from analysis because of predetermined exclusion criteria: arrhythmia (n = 2), technical error, or absence of epidural analgesia, atropine, phenylephrine need (Fig. 1). A total of 18 ASA 2–3 patients were included in the analysis (Fig. 1). The mean(SD) age was 62(13) years, the weight was 74(12) kg, and the length was 165(7) cm.

The average(SD) CO increased from 3.92(0.87) L min^−1^ at T_−1_ to 4.94(1.2) L min^−1^ at T_5_. Figure 2a–k shows the evolution in individual patients (thin lines) and average (thick line) values of the main hemodynamic variables during the period from 1 min before till 12 min after the increase in initial blood pressure. An overview of the chronicity and interactions of the mean values of all the investigated variables is comprehensively depicted in Fig. 3.

Changes in hemodynamics are summarized in Table 1. Between T_−1_ and T_5_, MAP, SV, CO, CVP and EtCO_2_ increased by 62, 28, 26, 33 and 11%, respectively, while both dynamic preload variables decreased: PPV dropped from 20% at T_−1_ to 9% at T_5_ and to 13% at T_10_ and SVV from 19 to 11 and 14%, respectively. Between T_−1_ and T_5_, the Pmsa increased by 37% and the Pvr by 41%. In addition, while the SVR increased by 37%, the RVR increased by 6% only.

**Table 1:** Evolution of the hemodynamic variables

	T_−1_	T_5_	T_10_	Δ(T_5_–T_−1_)	Δ(T_10_–T_−1_)
MAP (mmHg)	54(8)	88(16)*	67(12)*	34(12; 64)	13(2; 24)
HR (bpm)	72(10)	70(12)	70(11)	− 1(− 12; 7)	− 2(− 9; 5)
EtCO_2_ (mmHg)	38(4)	42(5)*	40(5)*	3(0; 7)	2 (0; 6)
CVP (mmHg)	8(5)	11(6)*	9(5)	2(− 1; 9)	0(− 2; 5)
CO (L min^−1^)	3.92(0.87)	4.94(1.2)*	4.71(1.23)*	0.9(− 0.08; 2.57)	0.55(− 0.31; 2.25)
SV (mL)	55(10)	70(14)*	67(14)*	17(3; 30)	9(− 1; 32)
PPV (%)	20(7)	9(5)*	13(5)*	− 11(− 21; − 2)	− 6(− 17; − 2)
SVV (%)	19(3)	11(6)*	14(6)*	− 8.5(− 15;3)	− 6(− 11; 1)
SVR (dyn s cm^−5^)	1035(305)	1421(499)*	1103(350)	370(80; 930)	80(− 110; 360)
Pmsa (mmHg)	13(4)	18(5)*	15(5)*	4(0; 12)	1(− 1; 7)
RVR (mmHg min L^−1^)	1.33(0.34)	1.54(0.45)*	1.38(0.38)*	0.09(0; 0.47)	0.02(− 0.06; 0.16)
Pvr (mmHg)	4.97(0.84)	7.02(1.24)*	5.97(1.04)*	1.79(0; 3.81)	0.84(− 0.13; 2.01)

Mean(SD) evolution of the hemodynamic variables: before administration of phenylephrine (T_−1_) and 5(T_5_) and 10 min (T_10_) after 10% increase in MAP. *P < 0.05 versus T_−1_. Median(range) changes in the hemodynamic variables between T_−1_ and T_5_, and between T_−1_ and T_10_

*MAP* mean arterial pressure, *HR* heart rate, *EtCO*_*2*_ end-tidal CO_2_ concentration, *CVP* central venous pressure, *CO* cardiac output, *SV* stroke volume, *PPV* pulse pressure variation, *SVV* stroke volume variation, *SVR* systemic vascular resistance, *Pmsa* mean systemic filling pressure, *RVR* resistance to vascular return, *Pvr* pressure for venous return

## Discussion

Phenylephrine is conventionally thought to negatively affect CO, or at best to have no influence if the cardiac contractility is able to overcome the increased afterload without loss of SV [10]. Our hypothesis, however, was on the contrary, that if a relative hypovolemia is present due to anesthesia-induced excessive vasodilation of the capacitance vessels, this could be corrected by phenylephrine, inducing an improved centralization of the available blood, eventually resulting in an increase in CO.

The main finding of this prospective study was that in patients with anesthesia-induced hypotension and preload dependency—defined as PPV > 12%, phenylephrine increases CO by virtue of an increase in return function. This is reflected in multiple distinct indices, all indicating an increase in CO, owing to a rightward shift in the position of the heart on the Frank–Starling relationship: the dissociation of MAP and SVR at T_1_, CVP, PPV, CO, and EtCO_2_.

In patients undergoing sigmoidectomy, a relative hypovolemia is common owing to the combination of several factors: the patients have been fasting from the night before, had bowel preparation, and received an epidural loading dose, combined with general anesthesia, all causing vasodilation.

This pharmacologically induced vasoplegia, together with gravitational venous pooling in the lower limbs due to the leg-down patient positioning—to prevent conflict with the surgeon’s arms—and an increase in abdominal pressure often results in a markedly decreased venous return [11]. While an increase in cardiac preload enhances the CO, recent evidence revealed that a zero-balance fluid approach is recommended in the elective perioperative setting to avoid adverse effects of unnecessary, excessive fluid administration [12, 13]. In bowel surgery, the additional concern for return of peristalsis, and oedema of the gut tissue emphasizes the importance of moderating fluid administration. Fluid restriction is therefore considered a part of the care package for enhanced recovery after colorectal surgery [14].

Importantly, this relative hypovolemic state is just of temporary occurrence. When surgery ends, and the patient awakens, the venous tonus and consequently CO will increase again spontaneously. As such, an alternative to enhance venous return, other than the irrevocable administration of fluids would be desirable. In those cases, the option of recruiting internal blood volume by increasing venous return through reversible pharmacological means such as vasopressors acting predominantly on capacitance vessels might be a potential alternative.

In an animal study, the average effect of phenylephrine on CO was related to the preload dependency of the heart: when the heart was preload dependent, phenylephrine induced an increase in CO [7]. Similarly, in a study with human patients, CO and SV decreased in preload-independent patients through an increase in cardiac afterload, but by virtue of increased venous return were unchanged in those that were preload-dependent [6].

In contrast to most studies analysing the combined effects of phenylephrine [3], its beneficial effect on the return function is much more pronounced in our patients where the combination of general anesthesia, leg-down position and epidural analgesia—which reduces alpha tone—induced distinctly different physiological conditions [15]. The actual blood flow in the body is determined by the intersection of the cardiac function and the return function, the latter defined by the stressed blood volume, of which the bulk is in the small venules and veins [16]. As such, the CO response to phenylephrine is very dependent on the starting condition of the return function: if the patient is volume replete, with good reserves in unstressed volume and minimal initial tone in the veins draining the compliant region, phenylephrine can recruit unstressed volume into stressed volume by contractions of the smooth muscles in the walls of the vessels of the compliant part of the circulation, increasing the venous elastic recoil pressure. When this effect is greater than the increase in venous resistance, this will result in increased venous return and CO [16].

Figure 3 and Table 1 show that 90 s after an initial parallel increase in MAP and SVR following phenylephrine administration, the SVR curve started to decrease steeply, while the MAP curve drops more slowly. This dissociation indicates a second phenomenon increasing the blood pressure independently of the vascular resistance system. Because MAP ~ CO × SVR, an increase in venous return offers an additional contribution to the effect of phenylephrine on the MAP. The onset of this effect—about 90 s following the initial increase in MAP—corresponds to the expected time to reach a significant concentration of phenylephrine in the venous capacitance vessels. Remarkably, at T_10_, the SVR is not significantly higher compared to T_−1_, while MAP and CO are still 24 and 20% higher, respectively (Table 1).

Next, the evolution of CVP—derived from the central venous catheter—also demonstrates a centralisation of venous blood, resulting in increased right ventricular preload, reflecting the postulated effects of phenylephrine on capacitance vessels. Even more, despite an increase in (left ventricular) CO—which depletes blood from the venous side—there is a persistent increase in CVP, implying that a higher cardiac preload is the primary driving force of the increased CO. This is also reflected in the steep increase in SV from 55 to 70 mL between T_−1_ and T_5_, despite the increase in systemic afterload, with no significant change in HR (Table 1). As surrogate measures of cardiac preload dependency, PPV—determined from the systolic and diastolic blood pressure measurements through the ventilation cycle—and SVV also distinctly drop. While PPV is not strictly a measure of preload, its decrease indicates a right-shift of the heart on the Frank–Starling relationship and thus a transition from preload dependence to fluid unresponsiveness without fluid administration [6]. Since all patients were preload dependent, no comparison in hemodynamic effects of phenylephrine between preload-dependent and preload-independent states was possible.

As a separate independent measure, the evolution of the EtCO_2_—measured by absorption spectrometry—indicates an increase in CO following phenylephrine [17, 18]. The significant average(SD) increase in EtCO_2_ from 38(4) to 42(5) mmHg during stable ventilatory settings and invariant HR also indicates an increase in CO owing to increased cardiac preload.

An important pharmacological consideration is the rather long biological half-life of phenylephrine of 2–3 h. While clinical experience based on the evolution of the MAP following phenylephrine administration gives the impression of a very short half-life, the fast decline of the MAP after the initial increase merely reflects the redistribution of the phenylephrine, but not its elimination. The long biological half-life of phenylephrine permits to reach a clinically significant plasma concentration in the capacitance vessels. While norepinephrine also has a potent α-mimetic effect, it has a biological half-life of only 2–6 min [19, 20]. Because of the fast enzymatic degradation of norepinephrine [20], the concentration in the capacitance vessels remains much lower—remind that the splanchnic capacitance vessels only receive a relatively small fraction of the CO, while harbouring 25% of the total blood volume [21]. Given these pharmacokinetic differences, the ratio of the effects on CO and MAP will arguably be more balanced following phenylephrine compared to norepinephrine administration. This should, however, be substantiated in further research.

This emphasizes the physiological complexity determining the ultimate effect of the used vasopressor on the CO: (1) the balance of α- and β-adrenoceptor effects, (2) the pharmacokinetic properties of the vasoactive molecule, and (3) the preload dependency of the patient.

Our study has several limitations: firstly, no echocardiographic measurements of diastolic right and left volumes were performed to assess the preload effects of phenylephrine. The used ProAQT/Pulsioflex device is yet to be formally validated as sufficiently accurate to measure the absolute values of the variables of interest. Although calibrated devices offer more accurate absolute values, the relative changes to baseline values can be acceptably described by pulse wave contour analysis technology for assessing trend changes within a moderate range of acceptable physiological values. Additionally, the accuracy of the calculated SV based on wave-contour analysis may have been affected by the change in arterial elastance following phenylephrine administration [22]. The evolution of the CO, however, is only one of several variables indicating an increase in cardiac filling due to phenylephrine. The particular advantage of this device based on fast-reacting algorithms is its high temporal resolution, averaging 4 “sliding” intervals of 7.5 s, which results in complete recalculation within 30 s [23]. Secondly, the patients were rather volume dependent due to the combination of vasoplegia by epidural analgesia and hypovolemia by bowel preparation, which may explain the distinct effects observed in this study, compared to other reports. Thirdly, atropine was administered at the start of anesthesia to attenuate the negative effects on the MAP and CO after induction with TIVA [24]. This doesn’t influence the effects of phenylephrine on the venous return, but it probably blunts the reflex bradycardia induced by phenylephrine. While this is beneficial to more clearly demonstrate the effects on the venous return and global hemodynamics, it may narrow the external validity of its beneficial effects on the CO. Fourthly, Pmsa and Pvr values were derived mathematically and therefore, coupled with CO. Ideally, these values should have been assessed independently of CO. In our study, this was not done because the inspiratory hold manoeuvres to measure Pmsa would have unavoidably disturbed the accuracy of the primary research variables. The algorithm to calculate Pmsa and Pvr was validated previously [25]. Finally, no radiographic confirmation of the tip of the central venous catheter was performed at the time of CVP measurement. While this would arguably have minor influence on the calculated changes to baseline values of the studied variables, it may have affected the accuracy of the CVP measurements.

With respect to the described limitations, the current results must be interpreted within the constraints of potential shortcomings. Neither preload, contractility nor afterload were directly measured by the pulse contour technology. While the observed results suggest an improvement of venous return by virtue of phenylephrine administration, further research relying on direct measurements, like echography or thermodilution will be needed to fully describe the changes in cardiac preload, contractility and afterload in these clinical conditions.

In this study, the evolution of the hemodynamic variables was investigated after injection of a single dose of phenylephrine. This strategy was selected in order to most reliably depict the chronology of the hemodynamic changes. Additional research investigating the hemodynamic effects of phenylephrine in different baseline preload states may further elucidate the preload-dependency of these compound effects. The effects during continuous infusion on the investigated variables will have to be precisely determined, as well as comparison with the effects of alternative vasopressors. Ideally, an independent measure of CO, such as ultrasound or thermodilution should have been used to confirm the observed evolution of waveform-derived variables.

### Clinical implications

The prospect to optimize cardiac preload with considerably less fluid administration offers significant clinical advantages but adds complexity due to differences in pharmacokinetics and patient characteristics. Excessive administration of vasopressors may jeopardize organ perfusion, which underlines the importance of advanced hemodynamic monitoring to assess the evolution of the hemodynamic variables for the guidance of the hemodynamic management. In summary, in preload-dependent patients with low SVR, vasopressors could be preferable, while in preload-dependent patients with high SVR, volume administration would be a better choice. Meticulous trend assessment of different indices of CO, volume responsiveness and SVR is imperative to individualize the optimal drug dose to maximise centralisation of blood while avoiding harmful effects on cardiac afterload and organ perfusion.

## Conclusions

This study indicates that in preload-dependent patients, phenylephrine increases the CO by virtue of an increase in cardiac filling. This is manifested by several distinct hemodynamic indices of CO and venous return, namely the dissociation of MAP and SVR, CVP, PPV, CO, and EtCO_2_, in addition to the derived variables SV, SVV, Pmsa, Pvr and RVR.
